# Construction Strategy for Flexible and Breathable SiO_2_/Al/NFs/PET Composite Fabrics with Dual Shielding against Microwave and Infrared–Thermal Radiations for Wearable Protective Clothing

**DOI:** 10.3390/polym16010006

**Published:** 2023-12-19

**Authors:** Hui Ye, Qiongzhen Liu, Xiao Xu, Mengya Song, Ying Lu, Liyan Yang, Wen Wang, Yuedan Wang, Mufang Li, Dong Wang

**Affiliations:** 1 Key Laboratory of Textile Fiber and Products, Ministry of Education, Wuhan Textile University, Wuhan 430200, China; huiye951021@163.com (H.Y.); tiananxing990808@163.com (X.X.); cherrysongmy01@163.com (M.S.); jingmenluying@163.com (Y.L.); yangliyan0427@163.com (L.Y.); 2020017@wtu.edu.cn (W.W.); wangdandan530@hotmail.com (Y.W.); mfli@wtu.edu.cn (M.L.); 2 Hubei International Science and Technology Cooperation Base for Intelligent Textile Material Innovation & Application, Wuhan Textile University, Wuhan 430200, China

**Keywords:** conductive fabrics, multi-band compatible shielding, electromagnetic shielding interference, infrared–thermal radiation, infrared stealth, thermal–moisture comfort

## Abstract

Microwave and infrared–thermal radiation-compatible shielding fabrics represent an important direction in the development of wearable protective fabrics. Nevertheless, effectively and conveniently integrating compatible shielding functions into fabrics while maintaining breathability and moisture permeability remains a significant challenge. Here, we take hydrophilic PVA-*co*-PE nanofibrous film-coated PET fabric (NFs/PET) as a flexible substrate and deposit a dielectric/conductive (SiO_2_/Al) bilayer film via magnetron sputtering. This strategy endows the fabric surface with high electrical conductivity, nanoscale roughness comparable to visible and infrared waves, and a dielectric–metal contact interface possessing localized plasmon resonance and Mie scattering effects. The results demonstrate that the optimized SiO_2_/Al/NFs/PET composite conductive fabric (referred to as S4-1) possesses favorable X-band electromagnetic interference (EMI) shielding effectiveness (50 dB) as well as excellent long-wave infrared (LWIR) shielding or IR stealth performance (IR emissivity of 0.60). Notably, the S4-1 fabric has a cooling effect of about 12.4 °C for a heat source (80 °C) and an insulating effect of about 17.2 °C for a cold source (−20 °C), showing excellent shielding capability for heat conduction and heat radiations. Moreover, the moisture permeability of the S4-1 fabric is about 300 g/(m^2^·h), which is better than the requirement concerning moisture permeability for wearable fabrics (≥2500–5000 g/(m^2^·24 h)), indicating excellent heat and moisture comfort. In short, our fabrics have lightweight, thin, moisture-permeable and excellent shielding performance, which provides novel ideas for the development of wearable multi-band shielding fabrics applied to complex electromagnetic environments.

## 1. Introduction

The current proliferation of wireless portable devices and high-frequency communication technologies has caused serious electromagnetic interference (EMI) to precision electronic components and communication systems, as well as potential threats to human health [1,2]. Therefore, the development of high-performance EMI shielding materials is critical [3,4]. X-band microwaves (8.2–12.4 GHz, 25–37.5 mm) are very prevalent in the surrounding environment and artificial equipment [5,6], and they have significant thermal effects on human tissue [7,8], thus becoming a priority band for microwave radiation shielding. Moreover, infrared shielding materials can mask the infrared characteristics of the target object under the infrared detector [9,10], achieving infrared camouflage, and they can also shield the thermal radiation emitted by high-temperature sources or objects, reducing the threat to human health, the environment and sensitive equipment. Due to the increasing complexity of electromagnetic environments in fields such as the electronics industry, aerospace and military, there is an urgent need to develop protective materials that can perform multi-band compatible shielding or compatible camouflage [11,12]. Furthermore, the application scenarios require the shielding materials to be flexible, lightweight and wearable. Fabrics have these advantages, as well as breathability, three-dimensional curved surface fit, and cuttability. Therefore, the development of fabrics with microwave and infrared–thermal radiations compatible shielding functionality for wearable protective apparel has attracted widespread attention.

The construction of multi-band compatible shielding functionality on the same fabric requires regulating the interaction between the fabric’s surface interface and electromagnetic waves of different wavelengths. Electromagnetic shielding prevents the propagation of electromagnetic waves in free space by using a shielding medium that reflects or absorbs high-frequency electromagnetic waves [13]. Infrared–thermal radiations shielding reduces or blocks the thermal radiations emitted by a radiation source or object using a shield [14]. Infrared radiation is an electromagnetic radiation with a wavelength range of 0.7–1000 μm. Typically, only mid-wave infrared (MWIR: 3–8 µm) and long-wave infrared (LWIR: 8–14 µm) radiations in the transparent window of the atmosphere can be detected using special equipment such as thermal imaging cameras or infrared thermometers [15]. According to Kirchhoff’s law of thermal radiation [16], the emissivity (ε) of a thermal radiation source is equal to its absorption coefficient (*α*), and both are related to its reflectance (*r*) and transmittance (*t*) according to the equation ε=α=1−r−t. To lower the emissivity of a thermal object, either *r* or *t* should be increased. Hence, decreasing the emissivity of the shielding medium’s surface in the mid-wavelength infrared (MWIR) and long-wavelength infrared (LWIR) regions can effectively reduce the thermal radiation contrast between the source and the ambient environment, achieving the infrared stealth of the source or preventing thermal radiation leakage from the high-temperature emitter [15]. Based on the above considerations, to achieve compatible shielding for microwave and mid-wave and long-wave infrared, a feasible approach is to increase the fabric reflectance for X-band electromagnetic waves and the MWIR (3–5 μm) and LWIR (8–14 μm) regions.

Metals exhibit high electrical conductivity and can reflect incident microwaves efficiently due to the collective oscillations of surface free electrons under microwave radiation, resulting from the skin effect. Moreover, the Hagen–Rubens law states that the emissivity of a metallic material in the infrared regions is inversely proportional to its electrical conductivity [17]. Therefore, metals are ideal IR and microwave shielding materials, and Al, Cu, and stainless steel are preferred for their outstanding performance and affordability. Moreover, various materials are also believed to selectively reflect or scatter electromagnetic waves in multiple bands, such as microwave and mid- to long-wave infrared. These materials include semiconductor oxides with high electrical conductivity (e.g., antimony-doped tin dioxide (ATO) [18]), two-dimensional layered materials with tunable bandgap (e.g., Mxene [19]), micro/nano-structured metamaterials with strong light–matter interactions (e.g., metal–dielectric–metal (MDM) film [20]), and high refractive-index dielectrics (e.g., SiO_2_ [21,22]). Therefore, current research efforts are mainly devoted to preparing highly conductive fabrics and constructing unique surfaces/interfaces for compatible modulation or manipulation of microwave and infrared waves. However, various nanostructured metals (e.g., silver nanowires) and two-dimensional layered materials, which are mainly combined or integrated with fabrics through processes such as spinning, impregnation, coating, or layer-by-layer assemblies [23], suffer from the limitations of high cost and poor performance stability. Moreover, metamaterials are typically periodic in structure, demand extremely precise lithography, self-assembly or 3D printing techniques, and are highly challenging to construct on fabric surfaces with very high roughness [24]. It is noteworthy that the most reliable and readily fabricated shielding materials are surface metallized fabrics prepared via various electroplating or chemical plating processes. Despite their advantages, these metallized fabrics are plagued by problems such as pollution during preparation, poor air/moisture permeability, reduced flexibility, and coatings that are prone to peeling. Moreover, the metal coating on the fabric surface undergoes oxidation and corrosion when exposed to air and moisture for a long time. This phenomenon increases the infrared emissivity on the fabric surface [25] and decreases the shielding effect significantly. Therefore, developing fabrics that can shield against microwave and infrared–thermal radiation across multiple spectrum bands, while maintaining their breathability and moisture permeability, remains a great challenge.

**Figure 1:**
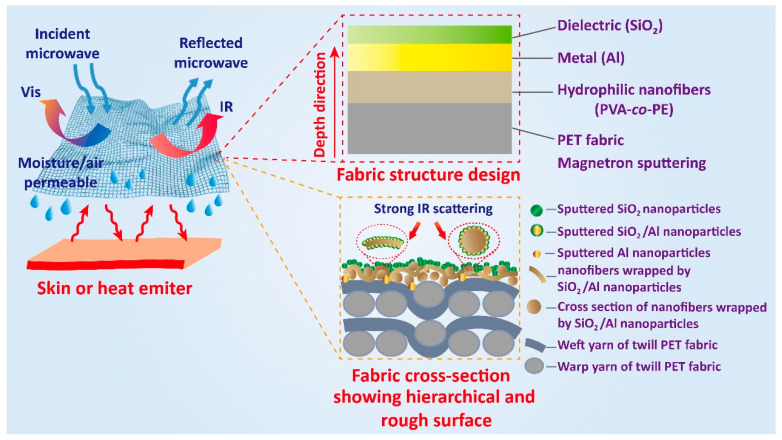
Scheme of the construction strategy and working mechanism of the flexible and breathable SiO_2_/Al/NFs/PET composite fabrics with dual shielding against microwave and infrared–thermal radiations.

This study adopts a strategy of constructing a dielectric/metallic (SiO_2_/Al) bilayer film structure on the surface of nanofiber-modified polyethylene terephthalate (PET) fabrics (denoted as NFs/PET) using the magnetron sputtering technique. As illustrated in Figure 1, this strategy endows the resulting SiO_2_/Al/NFs/PET fabrics with a compatible multiband shielding function against both microwave and infrared–thermal radiations, addressing the issues discussed previously. With this strategy, the nanofibrous membrane forms a three-dimensional (3D) connected hierarchical structural network on the PET surface, providing a continuous scaffold for the formation of a stable conductive network. Magnetron sputtering endows the SiO_2_/Al bilayer film with the characteristics of conformal growth, lightness, uniformity, and firm attachment on the fabric substrate [26,27], which can maximally preserve the texture structure and porousness of the fabric. Moreover, the SiO_2_/Al bilayers with surface microarray structures can effectively reflect or scatter IR and microwaves through a variety of interaction mechanisms, such as localized plasma resonance (LSPR), Mie resonance, dielectric–metal contact, and dielectric response. This study further establishes that the resulting SiO_2_/Al/NFs/PET multifunctional fabrics possess sound moisture permeability. In conclusion, this work provides innovative concepts and theoretical foundations for the development of wearable multi-band shielding fabrics for use in complex electromagnetic environments.

## 2. Experimental

### 2.1. Materials

The aluminum (Al) and silicon dioxide (SiO_2_) sputtering targets were purchased from Yingfeixun Optoelectronic Technology Co., Ltd. (Fuzhou, China). The anhydrous ethanol and isopropanol were obtained from Sinopharm Chemical Reagent Co., Ltd. (Shanghai, China). The twill PET fabrics with an average thickness of 330 μm (grammage of 179.5 g/m^2^) were purchased from Taobao.com. The PVA-*co*-PE nanofibers used in this study were prepared via the melt-extrusion phase separation method invented in our laboratory [28]. All the purified water was self-made in our lab.

### 2.2. Preparation Methods

Preparation of the NFs/PET hydrophilic fabric substrate. A total of 180 mL of a 1 wt.% PVA-*co*-PE nanofiber dispersion was prepared utilizing a pulper’s high-speed shear force, with a 1:1 solvent ratio of isopropanol to water and 1 mL of glutaraldehyde as a cross-linking agent [28]. Then, the nanofiber dispersion was evenly sprayed onto the surface of a twill PET fabric (15 × 15 cm^2^) via a spray gun, and the resulting modified fabric was designated as NFs/PET fabric with a thickness of 365 μm.

Preparation of the Al/NFs/PET surface metallized fabrics**.** A high-vacuum multi-target magnetron sputtering apparatus (TRP-450, Sky Technology Development Co., Ltd. Chinese Academy of Science, Shenyang, China) was used to sputter aluminum onto the NFs/PET fabric surface. The background vacuum (base pressure) in the sputtering chamber was 6.6 × 10^−4^ Pa. Before sputtering, the whole gas path was purged 2–3 times with flowing Ar gas and the target surface was pre-cleaned 2–3 times with plasma. During the sputtering process, the argon pressure was set at 0.3 Pa, the DC sputtering power was set at 80 W, and the distance between the target and the substrate was kept at 11 cm. Meanwhile, the substrate of the NFs/PET fabric did not need to be heated. For ease of recording, the fabrics with aluminum sputtering times of 5 min, 10 min, 15 min and 20 min were designated as S1, S2, S3 and S4, respectively.

Preparation of the SiO_2_/Al/NFs/PET surface functionalized fabrics. An SiO_2_ film was subsequently sputtered onto the surface of the S4 fabric described above. Before sputtering, an Ar-3% O_2_ gas mixture was used to purge the whole gas path 2–3 times, and plasma was used to clean the surface of the SiO_2_ target several times. The sputtering was conducted at a pressure of 0.5 Pa using the RF sputtering mode with a power setting of 120 W. The target–substrate spacing was still maintained at 11 cm, and the Al/NFs/PET fabric substrate was not heated. The fabrics with sputtered SiO_2_ films at 0, 30, 60, and 90 min are referred to as S4, S4-1, S4-2, and S4-3, respectively.

Preparation of the SiO_2_/Al/NFs/PET composite fabrics with a surface microstructure. Rectangular arrays of circular holes with diameters of 1 mm, 1.5 mm, 6 mm, 7.5 mm, and 9 mm were cut into a single sheet of A4 paper using a laser cutter. The above A4 paper containing circular holes was used as a sputtering template, tightly attached to the surface of the Al/NFs/PET substrate, and an SiO_2_ film was sputtered for 0.5 h to obtain the SiO_2_/Al/NFs/PET composite fabrics with different surface microarray structures. The resulting fabrics corresponding to the template apertures of 1 mm, 1.5 mm, 6 mm, 7.5 mm, and 9 mm were denoted as S4-1-1, S4-1-1.5, S4-1-6, S4-1-7.5, and S4-1-9, respectively.

It should be noted that the thickness of the film obtained via sputtering is difficult to measure due to the unevenness of the fabric substrate. Therefore, before depositing the films on the fabric, a smooth glass sheet was used as the substrate to create a step in the overlaying substrate method, and then the Al films and SiO_2_ films were deposited onto the substrate, respectively. The film thicknesses for the different deposition times were obtained via AFM scanning of these steps. The corresponding results are shown in Figure S1 of the Supplementary File, showing that the sputtering rate was about 19.54 nm/min for the Al film and 5.01 nm/min for the SiO_2_ film. Therefore, when the fabric was used as the sputtering substrate, the thickness of the Al film or SiO_2_ film sputtered on the fabrics could be evaluated using this sputtering rate since the other sputtering parameters were not changed.

### 2.3. Characterization and Testing

The macroscopic morphology and microstructure of the different fabric samples were observed using an ultra-deep 3D digital microscope (VHX-5000, KEYENCE, Osaka, Japan) and a scanning electron microscope (IT300A, JEOL, Tokyo, Japan). The surface morphology and roughness of the fabrics were characterized via the contact mode using an atomic force microscope (AFM) (Bruker Dimension Icon, Karlsruhe, Germany). The phase structure of the samples was determined utilizing an X-ray diffractometer (Empyrean, PANalytical, Almelo, The Netherlands). The reflectance and transmittance of the samples were measured via a UV–Vis–NIR spectrometry (SoildSpec-3700, Shimadzu, Kyoto, Japan) in a simulated integrating sphere mode with a wavelength range of 200–2500 nm. A BaSO_4_ white plate was employed for reference. The infrared emissivity of the samples was determined in the wavelength range of 2.5–25 μm at room temperature using a Fourier transform-infrared spectrometer (FT-IR) (Nicolet iS50, Thermo Fisher, Waltham, MA, USA) with an integrating sphere attachment. The infrared thermal images of the various fabrics were captured at different ambient temperatures utilizing an infrared thermal imager (Ti400, Fluke, Everett, WA, USA). Additionally, thermocouples (54IIB, Fluke) were used to monitor the composite fabrics’ actual temperature. The air and moisture permeability properties of all the fabric samples were individually tested with a fabric permeability tester (YG(B)461G) and a fabric moisture permeability meter (YG(B)216T), respectively.

The electrical conductivity of the diverse composite fabrics was evaluated using a fully automated four-point probe measuring instrument (RTS-9, 4Probes Tech Ltd., Guangzhou, China). The electromagnetic shielding effectiveness of the samples was measured in the frequency range of 8.2–12.4 GHz (X-band) using the waveguide transmission/reflection method with a vector network analyzer (ZNB20, R&S, Munich, Germany). Based on the Schelkunoff theory, the total shielding effectiveness (*SE_T_*) consists of the reflection loss (*SE_R_*), absorption loss (*SE_A_*), and multiple reflection loss (*SE_M_*), and it can be calculated as follows:

(1) $${SE}_{T}={SE}_{R}+{SE}_{A}+{SE}_{M},$$

(2) A+T+R=1,

(3) $${SE}_{T}=-10\log T=-10\log({s_{21}}^{2}),$$

(4) $${SE}_{R}=-10\log(1-R)=-10\log({{1-s}_{11}}^{2}),$$

(5) $${SE}_{A}=-10\log(T/(1-R))=-10\log(\frac{{s_{21}}^{2}}{{{1-s}_{11}}^{2}}),$$

where *S*_11_ and *S*_21_ are scattering parameters measured using a vector network analyzer, while *A*, *T*, and *R* represent the absorbed, transmitted, and reflected power coefficients, respectively. According to the Schelkunoff theory, *SE_M_* can be neglected when *SE_A_* is greater than 15 dB.

## 3. Results and Discussion

### 3.1. Macroscopic Morphologies and Microstructures

**Figure 2:**
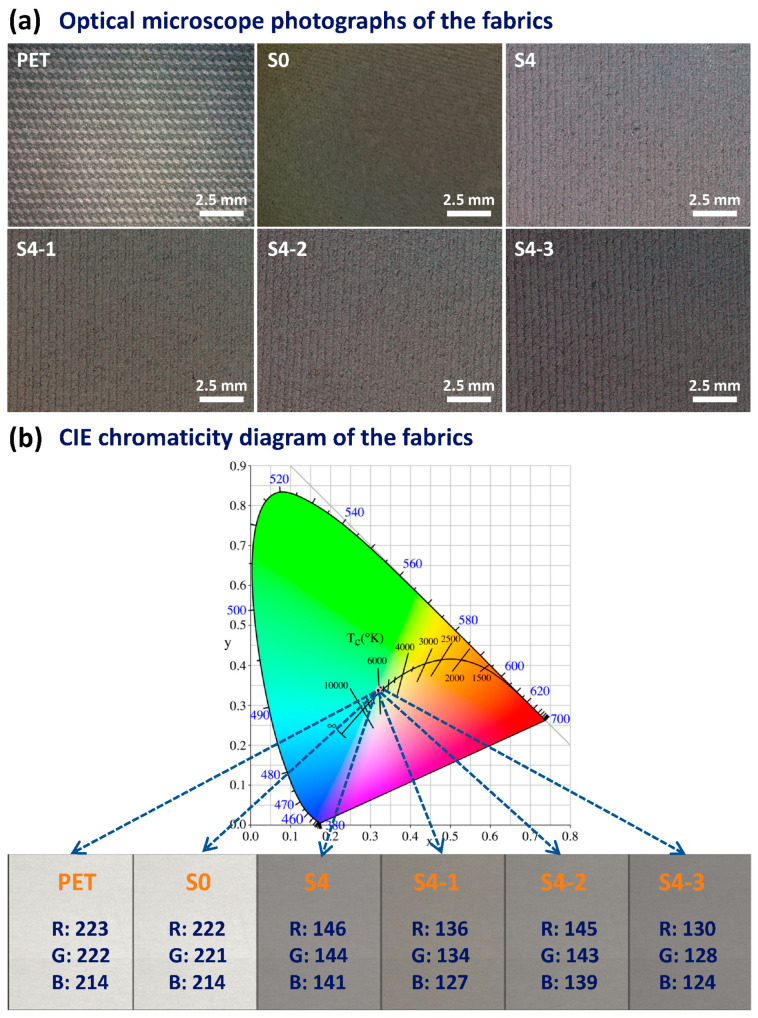
(**a**) Optical microscope photographs and (**b**) the CIE chromaticity diagram (with the corresponding RGB color values) of the different fabrics.

Figure 2 displays surface macroscopic optical microscope images of the various fabrics and their color characterization. Figure 2a reveals that the PET substrate fabric is a normal twill weave structured fabric. Meanwhile, the texture structure of the PVA-*co*-PE nanofibers (NFs)-coated PET fabric (denoted as NFs/PET or S0) is slightly blurred, which is associated with light scattering via the stray distribution of the nanofibers on the fabric surface. The textures of the S4-1 to S4-3 fabrics are clearer after magnetron sputtering compared to S0, implying an enhanced difference in the reflection/scattering of light from the concave and convex parts of the texture configuration after sputtering. This reflects the thin and conformal growth of the deposited Al and SiO_2_ films on the surface of the NFs/PET fabrics. Figure 2b illustrates the CIE chromaticity diagram for the various composite fabrics along with their corresponding color visualization, including the RGB values. The RGB values for the PET based fabrics correspond closely to beige at (223, 222, 214). S4 has a metallic silver–gray color with RGB values of (146, 144, 141) resulting from the surface’s Al film coating. As the SiO_2_ sputtering time increases, the color of S4-1 to S4-3 changes from silver–gray (136, 134, 127) to dark gray (130, 128, 124), but the overall color does not change much. To summarize, the color of the fabrics after sputtering varies from the original beige to a slightly metallic gray. This indicates that the surface of the composite fabrics significantly affects the reflection and scattering of visible light. Further details will be presented in the subsequent analysis of the UV–Vis–NIR spectra.

**Figure 3:**
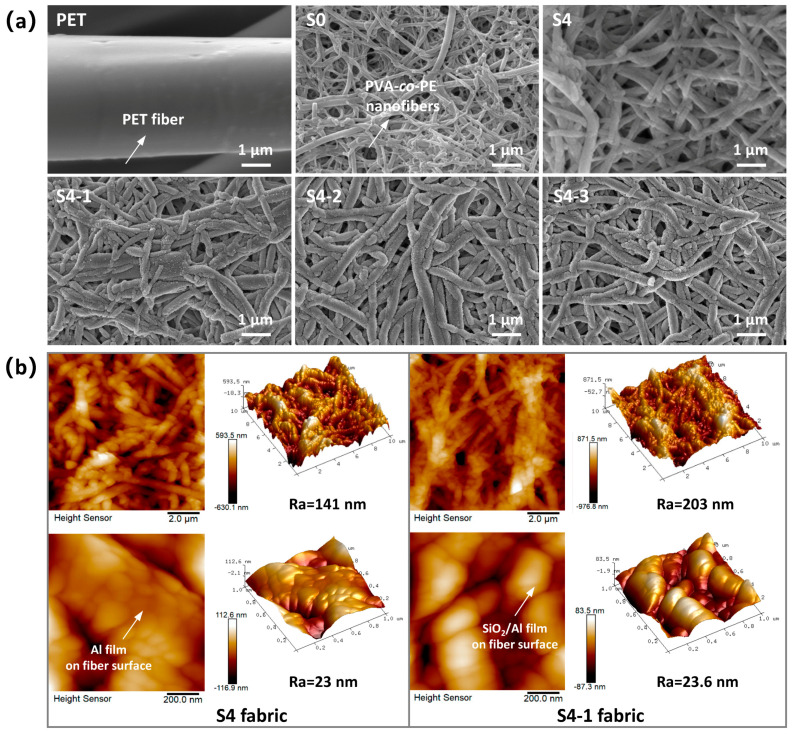
(**a**) SEM and (**b**) AFM microphotographs of the different fabrics.

Figure 3a displays SEM images depicting the surface microstructures of the various fabrics. The PET fabric exhibits a twill weave structure consisting of smooth fibers with a diameter of approximately 8 μm. In contrast, the surface of the NFs/PET (S0) fabric is coated with a film layer consisting of entangled nanofibers with a diameter of approximately 80–300 nm. The microstructure of the Al/NFs/PET (S4) fabric exhibits minimal difference from that of the S0 and a slight increase in the fiber diameter, likely due to the conformal growth of the Al film on the nanofiber surface. In addition, the diameter and roughness of the nanofibers on the surface of the S4-1 to S4-3 fabrics appear to increase significantly as the SiO_2_ sputtering time (0.5–1.5 h) increases, likely due to the thicker SiO_2_ film deposition. Figure 3b depicts the AFM morphologies and surface roughness of the S4 and S4-1 composite fabrics. The Al film on the S4 fabric comprises Al nanoparticles of about 50–200 nm, which are densely and tightly bound to the fiber surface. The fabric surface has an average roughness (Ra) of about 140 nm. After SiO_2_ film deposition, the surface roughness (Ra) of S4-1 increases to 203 nm. The SiO_2_ nanoparticles (150–300 nm) are coarser than the Al particles (50–200 nm) and are firmly bonded to the fabric surface. Overall, the Al and SiO_2_ films exhibit a very distinct feature of conformal growth on the surface of the NFs/PET fabrics. This feature is related to the mechanism of island or island-layer growth of the films during the magnetron sputtering process [29]. It can be speculated that nanofiber surfaces (raised regions) preferentially adsorb deposited atoms due to their higher surface energy and shorter deposition distances compared to inter-fiber gaps (recessed regions). The sputtered atoms continuously deposit onto the fiber surface, forming growth sites that stack into islands and expand in two and three dimensions. This process reconstructs a continuous film on the fabric surface, with texture characteristics similar to fabric.

### 3.2. UV–Vis–NIR Spectra and IR Emission Spectra

**Figure 4:**
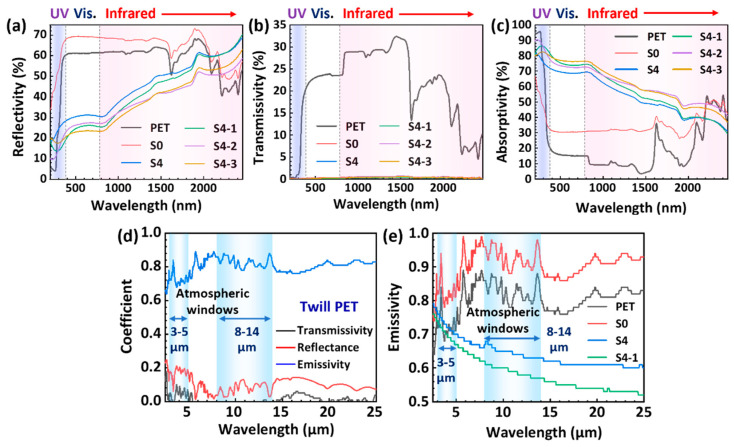
(**a**–**c**) UV–Vis–NIR spectra of the different fabrics and (**d**,**e**) their corresponding IR emissivities over the wavelength of 2.5–25 μm.

Figure 4a–c show the variation curves of the reflectance, transmittance and absorbance of the different fabrics in the UV–visible–NIR light bands. The neat PET fabric exhibits absorption-dominated properties for UV light at a wavelength of 200 nm (absorbance of 94.6%). As the wavelength increases, the PET fabric interacts with visible light (400–780 nm) and short-wave NIR light (800–1100 nm), mainly through reflection, with an average reflectance of 60%. In addition, there is a significant decrease in reflectivity and transparency and an increase in absorption in the long-wave NIR range (1100–2500 nm), particularly in the bands at 1624 nm, 2094 nm, 2217 nm, 2299 nm, and 2408 nm. This significant NIR absorption may be attributed to the first and second overtones of the stretching corresponding to hydrogen-containing groups (C–H groups sin aromatics molecules, -CH_2_ groups, –O–H groups) and oxygen-containing groups (–C=O, –C–O, –COO), as well as aromatic and methylene-based combination bands in the PET backbone [30]. Compared to the PET fabric, the NFs/PET fabric exhibits significantly increased reflectance (66–69%) and absorbance (30–32%) in the visible and short-wave NIR light regions, making it almost opaque. This improvement in reflectance can be attributed to the enhanced Mie scattering of visible and NIR light resulting from the hierarchical structured nanofiber membrane formed by the random entanglement of the PVA-*co*-PE nanofibers [31]. Furthermore, the thicker NFs/PET fabric has a higher absorption rate of visible–NIR light due to its reduced light permeability relative to the PET fabric. Notably, the reflectance of the Al20min/NFs/PET (S4) and SiO_2_/Al/NFs/PET (S4-1 to S4-3) composite fabrics drastically decreases to about 20–30% in the visible band, while the absorbance is enhanced to 70–80% and the transmittance is close to zero. This may be due to the micro/nano-scale roughness imparted to the fiber surface by the Al nanoparticles and SiO_2_ nanoparticles, which leads to the strong scattering and destructive interference of visible light [30]. Meanwhile, the reflectance of the Al/NFs/PET (S4) and SiO_2_/Al/NFs/PET (S4-1 to S4-3) composite fabrics rises from 23% to 70% with an increasing wavelength in the near-infrared region, while the absorbance reduces from 76% to 30%. Moreover, as the SiO_2_ sputtering time increases, the reflectance of the S4-1 to S4-3 composite fabrics declines and the absorption increases for visible and near-infrared light. These results are explicable through the application of the Hagen–Rubens law ($R\propto -4{\left(\upsilon \pi \varepsilon_{0}/\sigma \right)}^{1/2}$), which suggests that the reflectivity of a conducting material in the infrared spectrum is directly proportional to the conductivity of the material and the wavelength of the incident light.

Figure 4d presents the infrared emissivity (absorbance), reflectance and transmittance of the PET fabric in the 2.5–25 μm band. The PET fabric exhibits significant absorptivity in the MWIR, with an average value of about 0.76, whereas the reflectivity is below 0.2 and the transmittance is close to zero. Furthermore, the mean infrared (IR) emissivities of the PET fabric in the MWIR (3–5 μm) and LWIR (8–14 μm) IR imaging windows are 0.73 and 0.88, respectively. These results indicate that the twill PET fabrics exhibit black-body-like properties with strong absorption and emission characteristics of IR light and thus can be easily imaged using IR cameras. Figure 4e compares the IR emissivities of the four fabrics in the 2.5–25 μm band. The NFs/PET (S0) fabric exhibits higher average IR emissivities in the 3–5 μm and 8–14 μm bands (0.83 and 0.93) when compared to the PET. This may be attributed to the fact that the NFs/PET fabric has a greater thickness and denser pore size, which enhances multiple scattering and absorption. In contrast, the IR emissivities of the Al/NFs/PET (S4) and SiO_2_/Al/NFs/PET (S4-1) fabrics are significantly lower than those of the PET and NFs/PET. Specifically, the mean IR emissivity of the S4 fabric in the 3–5 μm and 8–14 μm bands is 0.73 and 0.65, respectively. The S4-1 fabric exhibits the lowest IR emissivity among all the fabrics, with average IR emissivities of 0.70 and 0.60 in the 3–5 μm and 8–14 μm bands, respectively. This can be attributed to the strong Mie scattering effect of the rough surface of the SiO_2_/Al composite nanoparticles in the IR band.

Taken together, the PET fabric has high visible light reflectivity (~0.6) and very high IR window (8–14 μm) emissivity (~0.88). Notably, the SiO_2_/Al/NFs/PET (S4-1) composite conductive fabric has a low visible light reflectivity (0.18–0.25) and infrared window emissivity (0.60), which are expected to realize excellent infrared stealth performance.

### 3.3. Infrared and Thermal Radiation-Shielding Properties

**Figure 5:**
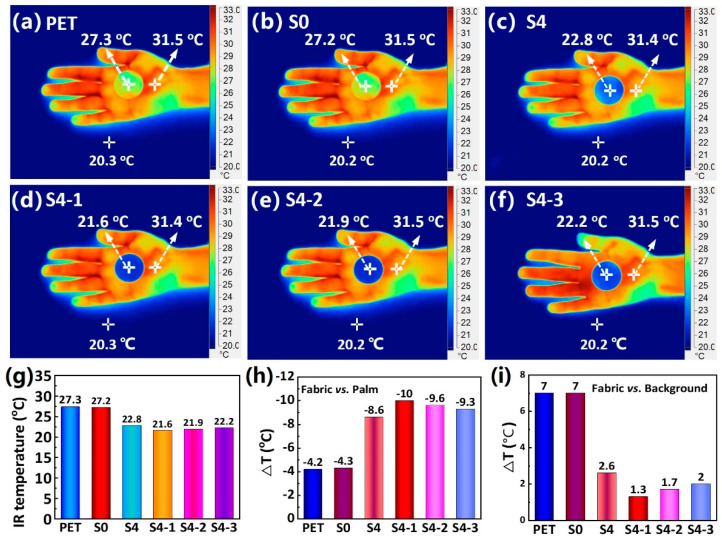
(**a**–**f**) IR thermograms of various fabric samples held on the palm and (**g**–**i**) IR temperatures at the center of the different fabrics, along with the difference in IR temperatures of the fabrics from the palm and background, respectively.

According to Planck’s law, any object with a temperature above absolute zero emits infrared light outwardly. An infrared camera’s detector captures this invisible radiation in the 3–5 μm and 8–14 μm bands, creating a thermal image. Therefore, variations in thermal imaging when using an infrared camera can be observed for objects with different surface thermal emissivities. Thus, if an infrared shielding material covers a thermally emitting target, the smaller the temperature difference in the thermal image between the covered target and the background, the greater the infrared camouflage effect of the shielding material. Figure 5a–e show infrared thermography photographs of different fabric samples placed in the palm. Additionally, to demonstrate the IR shielding effect of the fabrics, Figure 5g–i present bar charts depicting the IR temperatures at the centers of the different fabrics and their temperature variations with respect to the palm and the background, respectively. The IR temperature at the center of the PET textile is 27.3 °C, which is 4.2 °C lower than the IR temperature at the center of the palm and 7 °C higher than the IR radiation temperature of the background. These results suggest that the PET and NFs/PET fabrics are unable to provide effective infrared shielding for the human body due to their high IR emissivity. As noted, the IR temperature at the center of the Al20min/NFs/PET (S4) fabric is 22.8 °C, which is 8.6 °C lower than the IR temperature at the center of the palm. The difference from the background IR temperature is only 1.6 °C and displays an impressive IR shielding impact. It is worth mentioning that in comparison to S4, the S4-1 fabric has an even lower IR temperature at the center, with a difference as low as 1.3 °C from the background IR temperature. The exceptional IR shielding effect of the S4-1 fabric can be attributed to the high IR reflectivity of the SiO_2_/Al composite nanofilm on its surface. Furthermore, the reduction in the infrared shielding of the S4-2 and S4-3 textiles compared to the S1 textiles can be attributed to the enlargement of the SiO_2_ nanoparticles and the resulting weakening of the Mie scattering. In short, benefiting from the surface SiO_2_/Al composite nanofilms with low IR emissivity on the PET fabric, the SiO_2_-0.5 h/Al/NFs/PET (S4-1) composite fabric has excellent IR shielding performance.

To assess the shielding effectiveness of the fabrics against heat radiations from heat emitters with different temperatures, the fabrics were placed on a temperature control stage and the changes in the IR and actual temperatures of the fabrics were monitored simultaneously using an infrared camera and a thermocouple.

**Figure 6:**
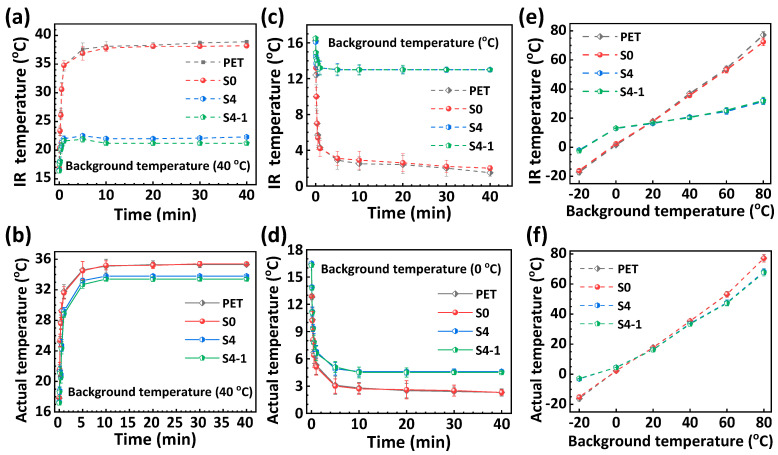
Change in both the IR and actual temperatures of the various fabrics as a function of time when placed on a temperature control stage at temperatures of (**a,b**) 40 °C and (**c**,**d**) 0 °C, and (**e,f**) variation in the IR and actual temperatures on the surface of the different fabrics as a function of the temperature of the background thermal emitter ranging from −20 °C to 80 °C.

Figure 6a,b depict the fluctuations in the IR and actual temperatures of the different fabrics as a function of the heating time when placed on the temperature stage at 40 °C. Specifically, the PET and NFs/PET fabrics display comparable characteristics in terms of the heat conduction and IR heat radiation, with their IR temperatures increasing rapidly to about 34.8 °C (actual temperature of about 31.6 °C) after 1 min and stabilizing at 36.9 °C (actual temperature of about 35.1 °C) after 10 min. These results indicate that the PET and NFs/PET fabrics have good thermal conductivity and can rapidly absorb and transfer heat from the surrounding environment (heat generator) to emit significant IR heat radiations. As observed, the IR and real temperatures of the SiO_2_-0.5 h/Al/NFs/PET (S4-1) fabric were slightly lower than those of the Al20min/NFs/PET (S4) fabric but much lower than those of the PET and NFs/PET fabrics. The thermal imaging temperature of the SiO_2_-0.5h/Al/NFs/PET (S4-1) fabric after 1 min was 21.6 °C (actual temperature of 28.8 °C), and the thermal imaging temperature was stabilized at 21.9 °C (actual temperature of about 33.4 °C) after 10 min. These results suggest that the SiO_2_-0.5 h/Al/NFs/PET (S4-1) fabric has a significant IR shielding effect against high-temperature (40 °C) IR emitters. Additionally, the S1 fabric’s temperature was 6.6 °C lower than that of the heat emitter and 1.7 °C lower than that of the ordinary PET fabric when exposed to continuous thermal conduction and thermal radiation from a 40 °C heat emitter. This confirms that the S1 fabric has a certain interfacial thermal resistance and a superior heat shielding capability for the propagation of high-temperature heat flow.

Figure 6c,d present the IR and actual temperatures of the different fabrics over time when the fabrics were placed on the temperature control stage at 0 °C. As observed, the IR temperatures of the PET and NFs/PET fabrics decreased immediately after contact with the cold stage and finally stabilized at 1.5 °C (actual temperature of 2.3 °C) after 40 min. Notably, the IR temperatures of the Al20 min/NFs/PET (S4) and SiO_2_-0.5h/Al/NFs/PET (S4-1) fabrics were stabilized at 13 °C after 10 min (the actual temperature is 4.5 °C). The results show that when in a low-temperature (0 °C) background environment, the SiO_2-_0.5h/Al/NFs/PET (S4-1) fabric has a slower cooling rate and a higher stabilized temperature after cooling (about 2.2 °C above) compared to the PET fabric. This is mainly due to the greater inward IR reflectivity and lower outward IR emission of the S4-1 fabric.

Figure 6e,f depict the changes in both the IR and actual temperatures of the various fabric surfaces with respect to the background temperature when placed in a variable temperature background (−20–80 °C). It should be noted that the temperature change within this range was carefully regulated using the temperature stage’s programmed setting and that each temperature point was allowed to stabilize for 30 min before heating up to the next temperature point. Overall, the infrared (IR) and actual temperatures of all the fabrics increased proportionally with the background temperature, which can be explained by the Stefan–Boltzmann law. It should be noted that when the PET and S4-1 fabrics were exposed to background temperatures below the test room temperature (20 °C), the actual temperatures were higher than the background temperature. Conversely, the actual temperatures of the PET and S4-1 fabrics were lower than the background temperature when exposed to background temperatures above the test room temperature (20 °C). For instance, when subjected to a low environmental temperature of −20 °C, the actual temperature of the S4-1 fabric (−2.8 °C) was 13.4 °C higher than the PET fabric (−16.2 °C) and 17.2 °C higher than the environmental temperature. Alternatively, when the background temperature reached 80 °C, the S4-1 fabrics displayed an actual temperature of 67.6 °C, which was 9.6 °C below the temperature of the PET fabric (77.2 °C) and 12.4 °C below the background temperature. These findings suggest that the S4-1 fabric has a higher interfacial thermal resistance than the ordinary PET fabric. Furthermore, the S4-1 fabrics exhibit cooling and insulating effects for hot and cold sources, respectively, indicating their exceptional heat shielding capabilities against both hot and cold sources. Overall, the S1 fabric exhibits excellent shielding against infrared and thermal radiation.

### 3.4. Electric Conductive Fabrics and Electromagnetic Shielding Effectiveness

**Figure 7:**
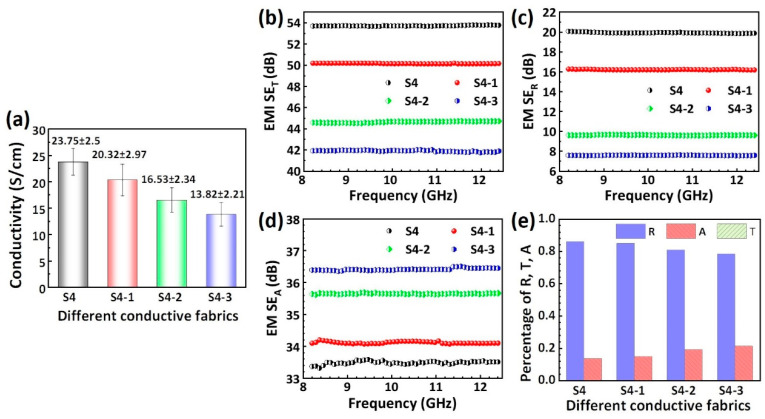
(**a**) Conductivity of the different electrical conductive fabrics and (**b**–**e**) electromagnetic shielding effectiveness of the fabrics in the X-band (8.2–12.4 GHz).

X-band electromagnetic waves within a frequency range of 8.2–12.4 GHz have high penetrative powers, pose the threat of electromagnetic interference (EMI) to electronic devices, and can adversely impact human health. Therefore, the shielding of this frequency band is crucial. Lightweight, portable, and wearable electromagnetic shielding fabrics offer an effective solution for protecting the human body and curved objects from EMI. Figure 7 shows the electrical conductivities of the different conductive fabrics and the electromagnetic shielding effectiveness of the fabrics in the X-band (8.2–12.4 GHz). As shown in Figure 7a, the electrical conductivity of the composite fabrics decreased gradually with the increase in the SiO_2_ sputtering time, from 23.75 S/cm for the S4 fabric to 13.82 S/cm for the S4-3 fabric. This result can be attributed to the dilution effect of the introduction of SiO_2_ dielectric films on the fabric conductivity. Figure 7b–d present the total electromagnetic shielding effectiveness (*SE_T_*), reflection loss (*SE_R_*) and absorption loss (*SE_A_*) of the different conductive fabrics in the X-band. The SE_T_ of S4, S4-1, S4-2, and S4-3 conductive fabrics were 53.8 dB, 50 dB, 45 dB, and 42 dB, respectively. It is observed that the shielding effectiveness decreases with the increase in the SiO_2_ content. The reflection loss (*SE_R_*) increased as the SiO_2_ content rises, paralleling the total electromagnetic shielding effectiveness. This trend can be primarily explained by the relationship ${SE}_{R}\propto \sigma^{1/2}$, that is, the reflection loss of the conductive fabric decreases with decreasing conductivity.

It is worth noting that the shielding mechanism of all the conductive fabrics against X-band microwaves in this study is primarily dominated by the reflection mechanism and supplemented by absorption. This is confirmed by the fact that the reflected power coefficients (R) (0.8–0.9) of the different conductive fabrics against X-band microwaves in Figure 7e are much higher than their absorbed power coefficients (A) (0.15–0.2). In addition, the contribution of the absorption loss to the total shielding effect increases slightly with increasing SiO_2_ content. This is due to the dielectric loss caused by interfacial polarization and dielectric polarization from SiO_2_ in the microwave field.

The stability and durability of the conductive fabrics when exposed to air and moisture need to be evaluated. For this purpose, we tested the electromagnetic shielding effectiveness of a piece of SiO_2_/AI/NFs/PET fabric (S4-1) at different relative humidities (40% to 80%) in the X-band, as shown in Figure S2 of the Supplementary File. The fabric was left in air for more than two months after preparation, but the total shielding effectiveness at constant temperature and humidity (RH = 37.2%, 16.2 °C) was still as high as 48.1 dB. The total electromagnetic shielding effectiveness of the fabric only decreased by about 8.3% when the relative humidity increased from 40% to 80%. After exposure to 80% RH, the fabric samples were allowed to naturally dry at room temperature for 1 h and still achieved a total shielding effectiveness of 45.65 dB, with a recovery rate of about 95%. This indicates that the overall performance of this S4-1 fabric sample in air and humidity is acceptable. However, the shielding performance of the fabric samples is susceptible to degradation when exposed to moisture for a prolonged period of time. Therefore, hydrophobic treatment of the fabric surface needs to be considered in our future research to improve the overall performance stability of the fabrics in service.

### 3.5. Influence of the Surface Microstructure

**Figure 8:**
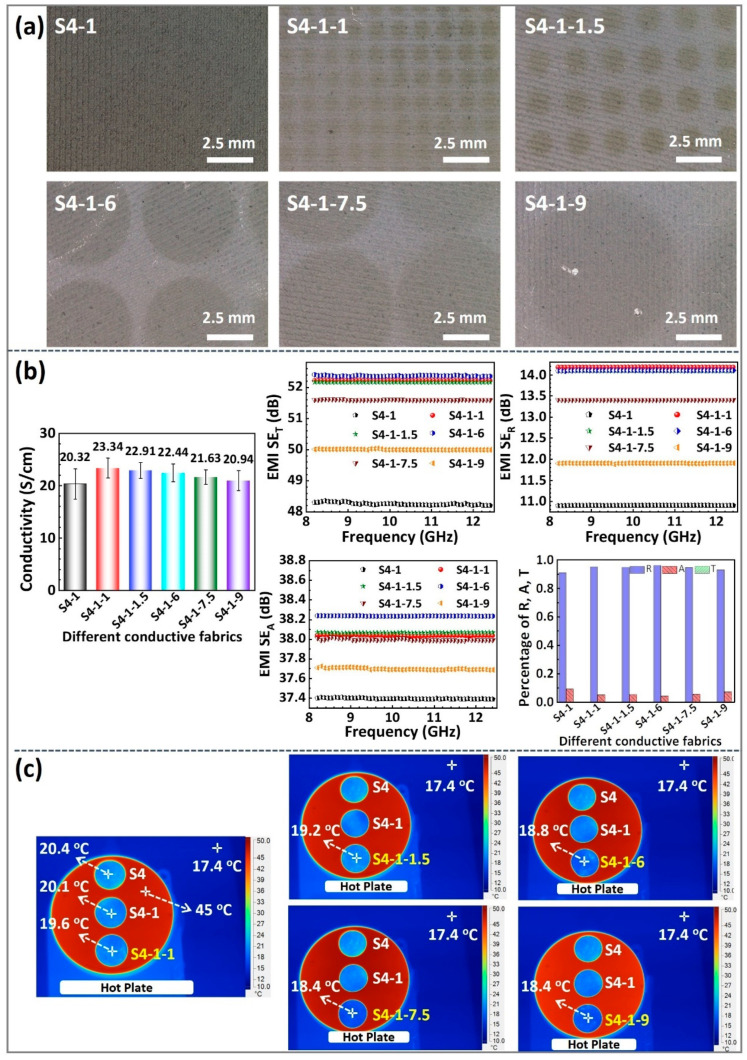
Various SiO_2_/Al/NFs/PET fabrics obtained using different sizes of mask plates: (**a**) the optical microscope photographs, (**b**) IR imaging photographs on a heated stage at 45 °C, and (**c**) electrical conductivity and electromagnetic shielding effectiveness.

It is known that the surface array structure of a thin film can change its scattering and emission characteristics with regard to electromagnetic waves by creating local or collective electromagnetic oscillation modes [32]. To achieve optimal IR and EMI shielding, sputtering techniques and circular-hole mask templates were utilized to generate a series of SiO_2/_Al/NFs/PET composite conductive fabrics with surface microstructures. The macroscopic optical morphologies, infrared imaging photos, and EMI shielding effects of these fabrics are presented in Figure 8. Notably, these composite fabrics have been assigned the names S4-1-1, S4-1-1.5, S4-1-6, S4-1-7.5, and S4-1-9 based on their circular hole template sizes of 1 mm, 1.5 mm, 6 mm, 7.5 mm, and 9 mm, respectively.

As shown in Figure 8a, arrays of gray dotted regions (resembling SiO_2_ islands) were generated on the optical image using a circular-hole mask plate, and the area of the region increased synchronously with the increase in the circular-hole size. Note that the light-colored regions between the gray dotted regions represent the un-sputtered SiO_2_ regions blocked by the mask plate, i.e., the conformally pre-deposited Al films on the NFs/PET fabric. In brief, the application of the circular-hole mask plate during the preparation of the SiO_2_/Al/NFs/PET composite fabric resulted in an SiO_2_ film with a microarray structure on its surface. This unique structure is expected to offer distinct response behaviors to different bands of electromagnetic waves compared to the S4-1 fabric with a uniform surface.

The electrical conductivity and electromagnetic shielding effectiveness of the SiO_2_/Al/NFs/PET composite fabrics with varying surface microstructures are displayed in Figure 8b. It is observed that the electrical conductivity of the S4-1 fabric, excluding the mask plate, was 20.32 S/cm. Nonetheless, the electrical conductivity of all the fabrics with a mask plate showed a slight increase. Meanwhile, the electrical conductivity of the SiO_2_/Al/NFs/PET composite fabrics decreased as the aperture size of the mask plate increased, ranging from −23.32 S/cm for the S4-1-1 fabric to 20.94 S/cm for the S4-1-9 fabric. This result is attributed to the fact that a larger template aperture results in a larger area covered by electrically insulating SiO_2_ islands. Additionally, the overall electromagnetic shielding effectiveness of the SiO_2_/Al/NFs/PET composite fabrics with various mask plates consistently decreased with an increasing template size from 52.22 dB to 48.3 dB, which is in line with the conductivity trend. Notably, only the S4-1-6 fabric exhibits distinct behavior. Among all the fabrics, it possesses the highest total electromagnetic shielding effectiveness (52.4 dB) despite having lower conductivity compared to S4-1-1 and S4-1-1.5. The unique features of the S4-1-6 fabric may be associated with the increased microwave scattering resulting from the 6 mm diameter of the array of SiO_2_ islands placed on its surface, which is closest to 1/4 λ of the X-band electromagnetic wave (8.2–12.4 GHz, 25–37.5 mm). The SiO_2_/Al/NFs/PET composite fabrics with varying mask plate sizes conform to the electromagnetic shielding mechanism in the X-band, with reflection being predominant (all R values greater than 0.9) and absorption acting as a supplement, as evidenced by the power coefficients of the reflection, absorption, and transmission. The reflection coefficient of the S4-1-6 fabrics is 0.96, which is higher than other conductive fabrics, owing to the impact of the surface array structure.

Figure 8c compares the IR thermograms of the SiO_2_/Al/NFs/PET composite fabrics that feature different surface microstructures after being stabilized at a heating table (45 °C). As shown, the IR temperature of the S4-1 fabric was 20.1 °C, whereas the IR temperatures of all the surface microstructured fabrics slightly decreased. It is found that the IR temperature of the SiO_2_/Al/NFs/PET composite fabrics decreased from 19.6 °C for S4-1-1 fabric to 18.4 °C for S4-1-9 fabric as the template size increased. For a better understanding, see the IR temperature variations between the fabric samples and the high-temperature stage in Table S1 of the Supplementary File in detail. The results indicate that the array structure comprised of SiO_2_ islands enhances the scattering of mid-wave IR to far-wave IR, thereby reducing the surface IR emissivity of the conductive fabrics. The structure also displays strong scattering ability for near-infrared light, as illustrated in Figure S3 in the Supplementary File. In short, as the size of the SiO_2_ islands in the array increases, the composite SiO_2_/Al/NFs/PET fabric surface provides improved IR shielding against thermal emitters.

In summary, SiO_2_/Al/NFs/PET composite fabrics with microarray structures on the surface can be achieved using a mask plate. Moreover, the surface microarray structure design has been demonstrated to increase the scattering ability of infrared and X-band electromagnetic waves, thus enhancing the composite fabric’s electromagnetic shielding effectiveness and shielding of infrared and thermal radiations.

### 3.6. Air Permeability and Moisture Permeability of the Composite Fabrics

The air and moisture permeability of fabrics has a major impact on the thermal and sensory comfort of the wearer [33] and is therefore a critical measure in the evaluation of high-performance wearable garments such as sportswear and protective clothing. Air permeability, in particular, determines the ease with which air passes through a fabric and is influenced by numerous factors, including the fabric density, structure, porosity, and coating. Moisture permeability is a measure of how easily water vapor can penetrate fabric and is related to both air and liquid moisture permeability.

**Figure 9:**
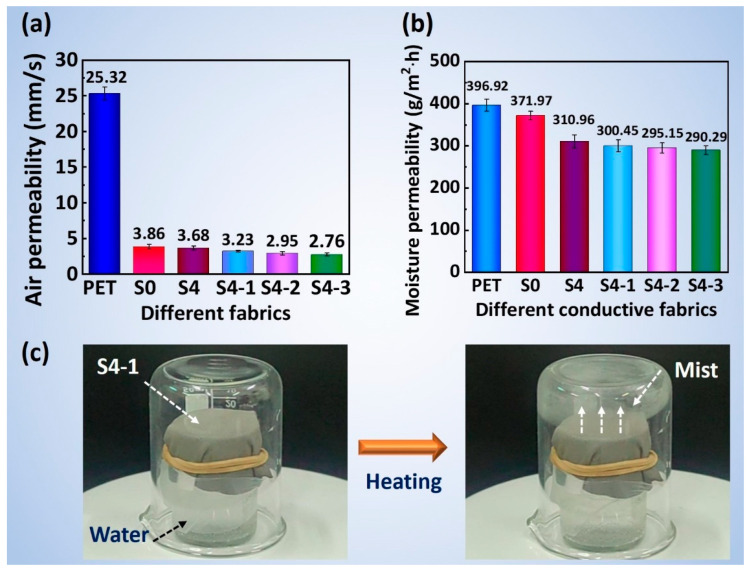
(**a**) Air permeability and (**b**) moisture permeability of the composite fabrics, and (**c**) visual photographs confirming the breathability of the S4-1 fabric.

Figure 9a demonstrates that the air permeability of the PET fabrics was 25.32 ± 0.93 mm/s, while the permeability of the S0 fabrics decreased to 3.86 ± 0.34 mm/s after being modified with NFs. This decrease is due to an increase in fabric thickness and a decrease in porosity resulting from the nanofibrous membrane on the fabric surface. The air permeability of the Al/NFs/PET (S4) fabric slightly decreased to 3.68 ± 0.27 mm/s in comparison to the NFs/PET(S0) fabric. This suggests that the sputtered films had a minimal impact on the overall thickness and porosity of the fabric. Moreover, after increasing the SiO_2_ sputtering time from 0.5 h to 1.5 h, the permeability of the SiO_2_/Al/NFs/PET composite fabrics decreased from 3.23 ± 0.12 mm/s for the S4-1 fabric to 2.76 ± 0.21 mm/s for 4-3 fabric. The reduction in air permeability is thought to be related to the increase in the surface thickness of the fabrics and the decrease in porosity, which prolonged the transmission path of the air flow. Moreover, all the fabrics in this study had an air permeability of less than 50 mm/s, rendering them suitable for use as protective clothing fabrics.

The moisture permeability of the PET, S0 and S4 fabrics were 396.92 ± 8.83 g/m^2^·h, 371.97 ± 7.36 g/m^2^·h and 310.96 ± 8.94 g/(m^2^·h), respectively, as shown in Figure 9b. As the SiO_2_ sputtering time increased from 0.5 h to 1.5 h, the moisture permeability of the SiO_2_/Al/NFs/PET composite fabrics slightly decreased from 300.45 ± 6.37 g/(m^2^·h) for the S4-1 fabric to 290.29 ± 8.73 g/(m^2^·h) for the S4-3 fabric. This decrease in moisture permeability is related to the longer free path of the H_2_O vapor molecules and the higher diffusion resistance due to the decrease in the number of air gaps and water channels in the composite fabrics. Notably, all the fabrics possessed moisture permeability greater than 2500 g/(m^2^·24 h), indicating good thermal and moisture comfort as protective clothing fabrics.

Combining the results concerning the air and moisture permeability of the fabrics, it is interesting to note that the air permeability of the NFs/PET fabric decreased by 80% compared to the PET fabric, but the moisture permeability remained at 94%. This result may be due to the high surface area and hydrophilicity of the PVA-*co*-PE nanofibrous membrane, which leads to the absorption and transport of water vapor through the capillary action of the nanopores. Additionally, the SiO_2_/Al/NFs/PET composite’s electrically conductive fabrics possess high moisture permeability due to the hierarchical porous structure of the NFs/PET fabrics and the advantages of magnetron sputtering film, such as conformality, light weight, and thinness. It is worth noting that Figure 9c visually depicts the moisture permeability of the conductive fabric. As shown, the S4-1 fabric covered the mouth of a small beaker, with the edge of the fabric in contact with the outer edge of the beaker being tightly sealed, and then the entire system was capped with a large transparent beaker. When the water in the small beaker was heated, the water vapor diffused upward through the fabric and cooled into a water mist at the top of the large beaker. Note that a dynamic video of water vapor passing through the S4-1 fabric is presented in Movie S1 (in the Supplementary File).

In addition, our SiO_2_/Al/NFs/PET conductive fabrics have asymmetric surface wetting properties toward water, with the PET side being hydrophilic and the SiO_2_/Al-coated side being hydrophobic, as shown in Figure S4 in the Supplementary File. Although the magnetron sputtered coating has a strong bond to the fabric substrate, the potential for the shedding of nanoparticles and surface layers and the resulting impact on human health and toxicity to the environment must be considered when used in high humidity environments [34,35]. Therefore, in practice, it may be necessary to consider waterproofing the entire fabric to eliminate potential hazards during use.

## 4. Conclusions

In summary, we have proposed a strategy to construct a dielectric/metallic (SiO_2_/Al) bilayer film structure on a nanofiber-modified PET fabric substrate to obtain a SiO_2_/Al/NFs/PET composite conductive fabric with a light weight, breathability, and dual shielding against microwave and infrared radiation. The composite conductive fabric is fabricated via the magnetron sputtering process. The manipulation of the fabric surface interaction with electromagnetic waves in the microwave and mid-infrared bands has been achieved by adjusting the fabric thickness, surface roughness and surface microarray structure. The optimized S4-1 fabric demonstrates excellent reflection-dominated EMI shielding effectiveness (50 dB) against X-band electromagnetic waves, while showing superior IR shielding/stealth capability (IR emissivity as low as 0.60) against LWIR. In addition, the fabric exhibits outstanding thermal shielding against both heat (80 °C) and cold (−20 °C) sources. Moreover, the S4-1 fabric has a moisture permeability of about 300 g/(m^2^·h), which ensures favorable heat and moisture comfort as a protective clothing fabric. Notably, the composite fabrics developed in this study are versatile and scalable, as they can be further optimized by adjusting the materials and ratios of the two dielectric/metallic layers. It is also foreseeable that flexible and breathable multi-band shielding or colorful invisible fabrics can be achieved via the effective manipulation of multi-band electromagnetic waves from visible light to microwaves using the fabric surface. Moreover, the fabric coating process can be easily industrialized using commercialized continuous automated magnetron sputtering equipment. Therefore, this work presents a novel and effective strategy for the development of multi-band compatible shielding fabrics, showing potential applications in electromagnetic protective clothing, stealth fabrics, and thermal and moisture comfort fabrics.

## Data Availability

Data will be made available on request.

## Supplementary Materials

The following supporting information can be downloaded at: https://www.mdpi.com/article/10.3390/polym16010006/s1.
