# Supplementary material for: Construction Strategy for Flexible and Breathable SiO2/Al/NFs/PET Composite Fabrics with Dual Shielding against Microwave and Infrared–Thermal Radiations for Wearable Protective Clothing
Source: Polymers (Basel). 2023 Dec 19;16(1):6. doi: 10.3390/polym16010006 (PMC10781071; doi:10.3390/polym16010006)
Supplement: Supplementary file 1 [file polymers-16-00006-s001.zip › polymers-2736996-supplementary.pdf]

Supplementary File

Construction strategy of flexible and breathable SiO_2/Al/NFs/PET composite fabrics with dual shielding against microwave and infrared-thermal radiations for wearable protective clothing

Hui Ye [1,2], Qiongzhen Liu [1,2*], Xiao Xu [1,2], MengYa Song [1,2], Ying Lu [1,2], Liyan Yang [1,2], Wen Wang [1,2], Yuedan Wang [1,2], Mufang Li [1,2], Dong Wang [1,2*]

[1] Key Laboratory of Textile Fiber and Products, Ministry of Education, Wuhan Textile University, Wuhan 430200, China

[2] Hubei International Science and Technology Cooperation Base for Intelligent Textile Material Innovation & Application, Wuhan Textile University, Wuhan 430200, China

***Corresponding Author**: Qiongzhen Liu, windlqz_2000@163.com; Wang Dong, wangdon08@126.com

List of Figures:

Figure S1. Changes in thickness and surface roughness for (a-b) Al films and (c-d) SiO_2 films sputtered on a glass substrate with respect to sputtering time.

Figure S2. Influence of the relative humidity on the electromagnetic shielding effectiveness of the S4-1 fabric: (a) SE_T, (b) SE_R, and (c) SE_A.

Table S1. IR temperature variations between the test fabric specimens and the hot plate center.

Figure S3. UV-Vis-NIR spectra of the various SiO_2/Al/NFs/PET fabrics with different surface arrays: (a) reflectivity, and (b) transmissivity.

Movie S1. A movie showing water vapor in a closed container penetrating through a SiO_2/Al/NFs/PET fabric, proving the breathability of the fabric.

Figure S4. The SiO_2/Al/NFs/PET conductive fabric (S4-1) showing asymmetric surface wetting properties toward water with (a) hydrophilic PET side and (b) hydrophobic coating side.

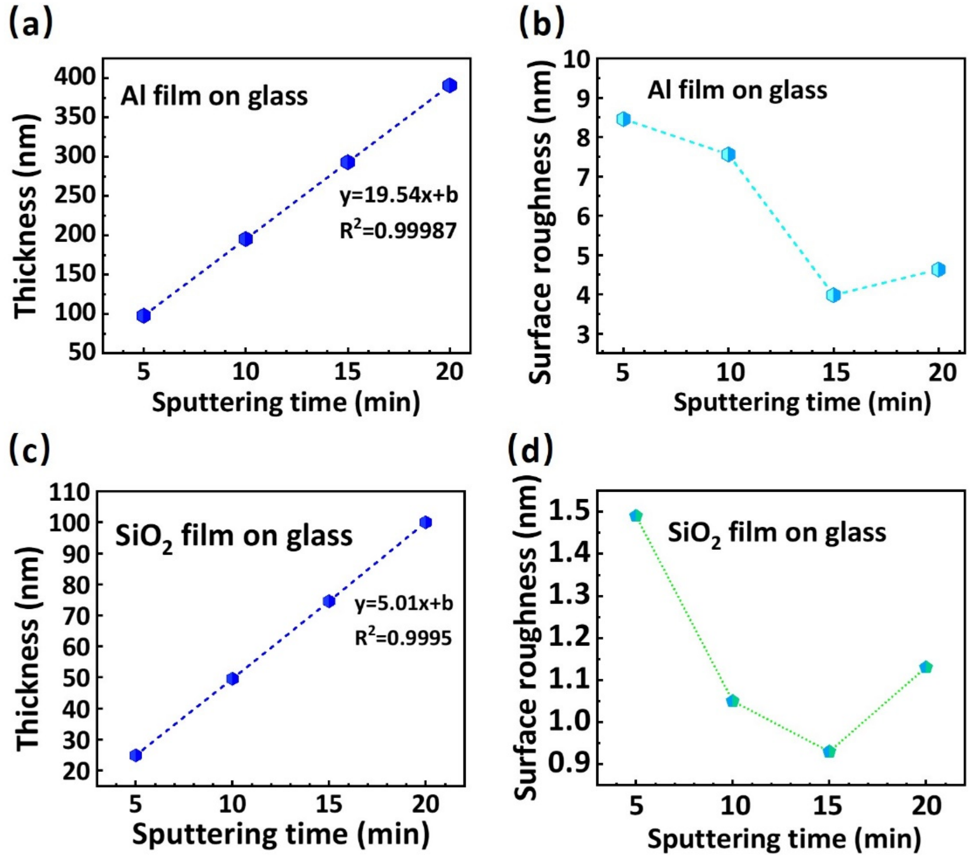

Figure S1. Changes in thickness and surface roughness for (a-b) Al films and (c-d) SiO_2 films sputtered on a glass substrate with respect to sputtering time.

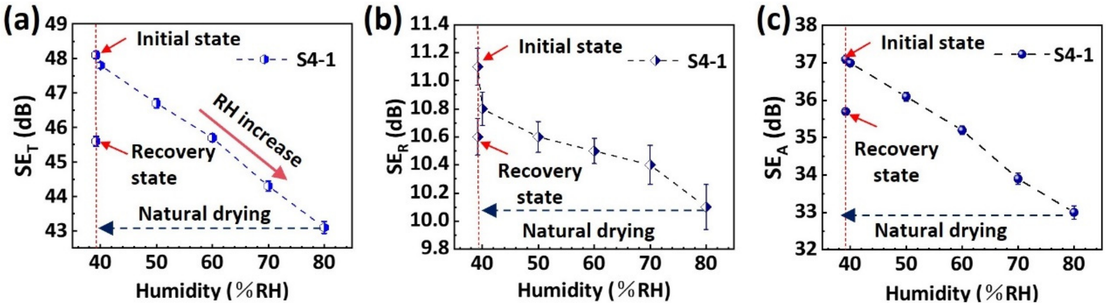

Figure S2. Influence of the relative humidity on the electromagnetic shielding effectiveness of the S4-1 fabric: (a) $SE_{T,}$ (b) SE_R, and (c) SE_A.

Table S1. IR temperature variations between the test fabric specimens and the hot plate center.

Fabric samples	IR temperature of samples (°C)	Hot stage temperature (°C)	ΔT (°C)
S4	20.4	45	-24.6
S4-1	20.1		-24.9
S4-1-1	19.6		-25.4
S4-1-1.5	19.2		-25.8
S4-1-6	18.8		-26.2
S4-1-7.5	18.4		-26.6
S4-1-9	18.4		-26.6

Note: ΔT denotes the IR temperature variation between the test fabric specimen and the hot plate center

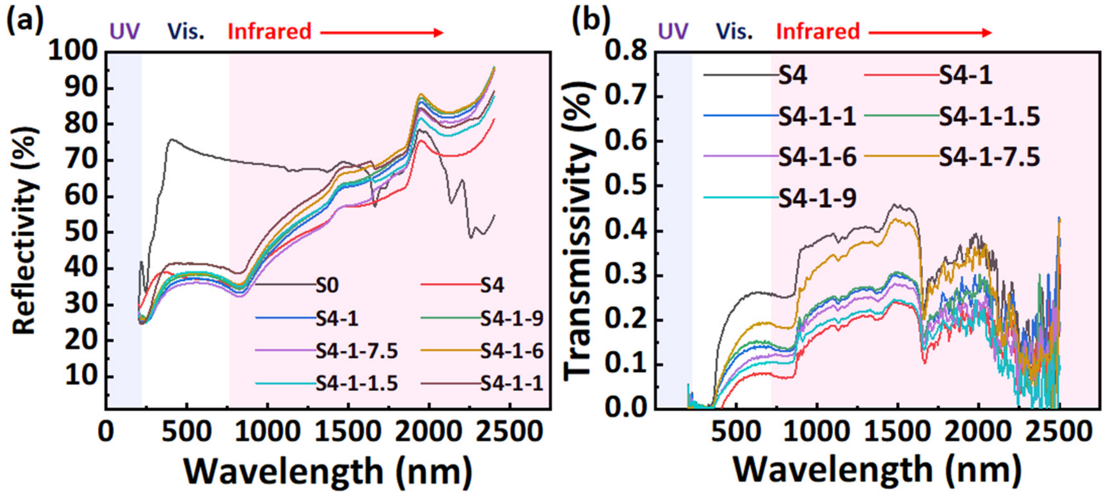

Figure S3. UV-Vis-NIR spectra of the various SiO_2/Al/NFs/PET fabrics with different surface arrays: (a) reflectivity, and (b) transmissivity.

Movie S1. A movie showing water vapor in a closed container penetrating through a SiO_2/Al/NFs/PET fabric, proving the breathability of the fabric.

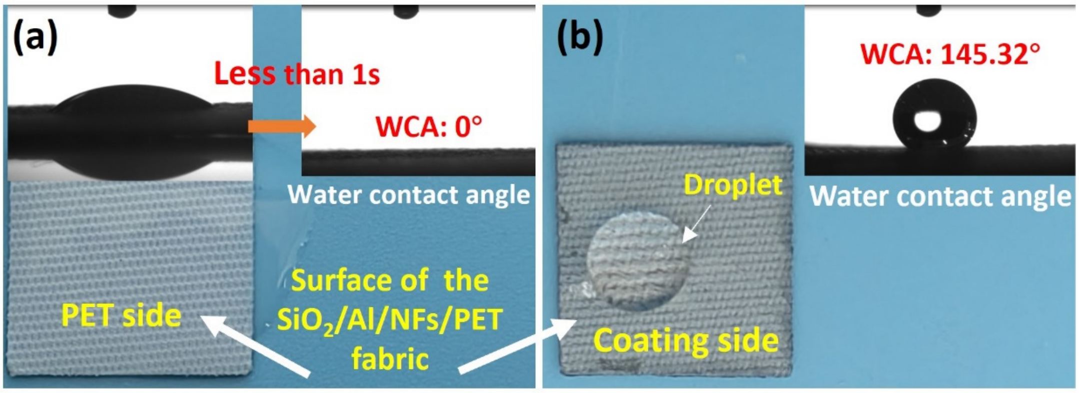

Figure S4. The SiO_2/Al/NFs/PET conductive fabric (S4-1) showing asymmetric surface wetting properties toward water with (a) hydrophilic PET side and (b) hydrophobic coating side.
